# The implementation of large-scale health system reform in identification, access and treatment of eating disorders in Australia

**DOI:** 10.1186/s40337-021-00476-8

**Published:** 2021-09-28

**Authors:** Sarah Maguire, Danielle Maloney

**Affiliations:** 1 InsideOut Institute for Eating Disorders, The University of Sydney, Sydney, Australia; 2 Sydney Local Health District, NSW Health, Sydney, Australia

**Keywords:** Health system change, Health reform, Mental health redesign, Workforce development, Multidisciplinary treatment, Eating disorders, Mental health system

## Abstract

**Background:**

It seems to be a truth universally acknowledged that pathways to care for people with eating disorders are inconsistent and difficult to navigate. This may, in part, be a result of the complex nature of the illness comprising both mental and medical ill-health across a broad range of severity. Care therefore is distributed across all parts of the health system resulting in many doors into the system, distributed care responsibility, without well developed or integrated pathways from one part of the system to another. Efforts in many parts of the world to redesign health service delivery for this illness group are underway, each dependent upon the local system structures, geographies served, funding sources and workforce availability.

**Methods:**

In NSW—the largest populational jurisdiction in Australia, and over three times the size of the UK—the government embarked six years ago on a program of whole-of-health system reform to embed identification and treatment of people with eating disorders across the lifespan and across the health system, which is largely publicly funded. Prior to this, eating disorders had not been considered a ‘core’ part of service delivery within the health system, meaning many patients received no treatment or bounced in and out of ‘doorways’. The program received initial funding of $17.6 million ($12.5 million USD) increasing to $29.5 million in phase 2 and the large-scale service and workforce development program has been implemented across 15 geographical districts spanning almost one million square kilometres servicing 7.75 million people.

**Conclusions:**

In the first five years of implementation there has been positive effects of the policy change and reform on all three service targets—emergency departments presentations, hospital admissions and community occasions of service as well as client hours. This paper describes the strategic process of policy and practice change, utilising well documented service design and change strategies and principles with relevance for strategic change within health systems in general.

## Plain English summary

This review outlines a $30 million health system reform to eating disorder treatment implemented by the NSW State Government six years ago which has seen large-scale service and workforce development across 15 geographical districts. This spans almost one million square kilometres and services 7.75 million people in Australia. The reform is very large in scale and is now in its second phase of implementation. Here outlined is the strategic process of policy and practice change of the entire reform and initial findings from an external review of phase one, which demonstrates positive effects on all three service targets—emergency departments presentations, hospital admissions and community occasions of service—including increased rates of treatment provision, improved perception of eating disorders amongst health professionals, improved pathway options and better communication within multidisciplinary teams. This type of whole-of-health system government led reform has relevance and learnings for health systems internationally.

## Background

“Accept that the development of clinical services is a long-term, organic and relationship-based business that must allow frequent and (effectively) continuous conversation between commissioners and providers to stand any chance of success”

*Tackling Whole-Systems Change: The Trafford Framework for Integrated Services*, Connor & Kissen, 2010 [1]

## Service development in eating disorders

Eating disorder (ED) service development on a large scale has been occurring in multiple places across the world. The U.K. have in the last five years funded national service development initiatives for EDs, including child & youth specialist multidisciplinary teams around the country [2, 3]. In the US where the national health system is fundamentally different and more fragmented than countries like the UK a national consensus document on the path forward for national treatment provision for EDs is about to be published [4]. And in Australia there have been multiple simultaneous large-scale efforts underway, both at a national level [5] and in a number of state jurisdictions [6, 7]. All acknowledge high levels of unmet need in terms of health system capacity and treatment provision for people with EDs. All cite system wide obstacles and the need for leadership, planning and organisation to redesign and implement changes to the health system. All outline a set of approaches unique to the setting and systems to achieve reform for this illness group. This paper outlines a health system reform program implemented in the largest government jurisdictional area in Australia, spanning almost one million square kilometres, servicing 7.75 million people and including multiple health districts. Many of the same challenges and strategic priorities confronted and used in other countries and states informed this reform program, and its implementation has broader implications for anyone undertaking service development in this illness group.

## The Australian health care system

Like most western countries, Australia utilises a complex health care system model involving large public and private sectors, inclusive of NGO (non-government) and NFP (not-for profit) organisations. Within the public health system both state and federal government-controlled services exist; within the private many different providers service all parts of the secondary and tertiary care spectrum, with limited but growing provision also in primary care [8]. The state led and funded public system is usually regarded as the doorway into hospital and other forms of secondary care, with private hospital services governing more as an alternate for those who have insurance cover rather than as a primary route.

The gatekeepers for health care, general practitioners (GPs) in primary care, reside outside of both the hospital and private systems and form part of federally funded and controlled health care. For most Australians this is the most frequently utilised health pathway. Hence, even at the earliest stages of health pathways into the system issues of integration, pathway coordination and continuum of care management arise.

In fact, use of the word ‘system’ implies the connections and integration of parts that oftentimes does not exist. It may be fairer to describe the Australian health system as a ‘set of systems’, which when working well integrate and transition seamlessly between services and when under-developed respond in their singular parts, able to do only what they are trained and equipped to do with little reference to other parts and capabilities of the system. This is especially so for mental health which relative to medical health receives a much smaller level of funding in Australia [9]- and in most countries around the world [10]- and is without the sub-speciality departments and networks that have been developed over centuries for the medical illnesses.

Many attempts have been made to improve the mental health care system in this country and others, with limited success [11]. This has led some to use the phrase the ‘re-disorganisation’ of mental health [12] to describe attempts to design the mental health ‘system’ in Australia to work better for those it serves.

Lived experience of health care within the health system overwhelmingly reflects this truth [13]. People with EDs, and mental illnesses more generally, receiving care within the Australia health system report numerous barriers to receiving effective treatment and treatment approaches that fail to account for the experience of the person receiving care [14].

As a result, reform within the mental health system that prioritises changing the user experience and getting effective evidence-based treatment to people faster, must be as much focused on building the system, creating connections, networks, pathways and coordinating care, as it is on funding and developing new services. There persists a preference for governments and health departments to make funding announcements tied to novel and innovative new services, but often patient experience and outcomes are better served by working with the existing parts of the system to build a cohesive system of care with ‘no wrong door’. There is evidence from whole-of-health system reforms that re-design and integration have proven more effective and cost-effective than procurement and single service funding [1].

## Eating disorder care within the health system

The illness itself is one of the greatest challenges to appropriate, consistent and effective care for an ED within a fragmented health system. The core features of fear and ambivalence are known barriers [15], which along with the complexity of EDs as both medical and mental health illness means there can be disagreements about where people should be managed and who should oversee their management [16].

Prior to the reform in this large jurisdiction of Australia, with no formal protocols for handling people with EDs in the hospital system, people with even severe illness reported being unable to gain access to hospital or repeated presentations to emergency before treatment was received [17]. As well as causing distress and anxiety for people with the illness and their carers, revolving door hospital encounters are very costly [18]. In hospital wards for those that did get admitted, clinical variability was the rule rather than the exception. The not uncommon experience in mental illness more broadly, where severely ill patients are shuttled between services; emergency departments, brief hospitalisations, and discharged without proper treatment and continuity of care [19] was very true of EDs. Children and adolescents were more likely to receive acute hospital admission than adults but were often discharged with no follow-up [17].

Community mental health teams in the majority considered EDs ‘out of scope’, in fact a lot of mental health services and structures available for the other major mental illnesses did not consider EDs as within scope [20]. There was very little delivery of evidence-based community treatments outside of city-based, small, specialist teams [17] meaning many people were unable to access a treatment with any chance of effectiveness outside of major cities. There was almost no treatment at all for bulimia nervosa (BN) or binge eating disorder (BED) within the public system and public health staff reported they felt unskilled and unwilling to treat the illnesses [21, 22], further contributing to services being reluctant to consider them as part of their core business.

Extrapolating from the best available epidemiological data for eating disorders within Australia [23, 24] and utilising 2014 census data there were in NSW, at the time the reform was announced, approximately 466,240 people with clinically significant eating disorders (AN 30,080 (0.4%), BN 82,720 (1.1%), BED 112,800 (1.5%), Other Specific Feeding and Eating Disorders 240,640 (3.2%)). From July 2013 to June 2014, 1370 individuals had contact with an ambulatory (outpatient) service within the public health system in NSW, which translates to approximately 0.2% of people with a clinically significant eating disorder accessing ambulatory mental health services. Other mental illnesses have at minimum 1–2% or more of the population with the illness recorded as accessing public mental health services, indicating a significant inequity in service provision for this illness group within the NSW health system [25]. Eight hundred and thirty-seven (837) individuals with either a primary or secondary diagnosis of an eating disorder received a hospital admission in the same period (2013–2014), 209 (28%) of these were hospitalised at one of the specialist hubs (150 child & adolescent hub; 59 adult hub) with the remaining admitted to general wards across the state [25]. One hundred and twenty-two people (122) made a presentation to an emergency department (25). While appropriate benchmarks for adequate rates of hospitalisation and emergency department treatment are more difficult to determine than for ambulatory care, these rates compared with incidence were considered low and the total number of people receiving care for an eating disorder in any one year within the public health system, relative to incidence, extremely low. It was acknowledged at the time that adequate recording of people with eating disorders accessing the health system was an issue, and improved recording was indeed a target of the reform, hence all data regarding presentations to the system need be regarded as indicative and likely an underestimation. Nonetheless, the case for urgent reform was clear.

## The process of strategic development

### Informed reform: lived experience and clinically led change

Prior to the announcement in late 2013 of the reform to the system of care for people with EDs in NSW, an advisory group comprising expert clinicians, people with a lived experience of an ED, academics and public health planners was convened by NSW Health. A project officer with public health qualifications was funded to map existing services, complete a needs analysis and review successful strategies for reform within health sectors. The committee met over a period of years to guide this process, review all outputs and develop a model for whole-of-health system change for ED pathways. During, this time they and others within their networks were building the case for change, advocating at the local level, to health system managers and administrators, to members of parliament (MPs), the health department and at times within the media.

### Advocacy and political will

The scoping of ED services and needs was supported and funded by NSW Ministry of Health, the department that oversees administration of state health services. Hence the program had both legitimacy and access to decision makers from the outset, both of which were key contributors to policy success. This strategic set-up was itself the result of innumerable years of agitation, conversations and advocacy to government by families, specialist clinician’s and academics. Ultimately it was the professional relationship between a senior clinician and the newly appointed director of the Ministry that saw the advisory committee convened and project officer appointed, highlighting the need for stakeholders in change to be alive to, and seize, opportunities at the moment they arise, and to understand and utilise the well documented role relationships play in policy change processes [26].

Once the plan was developed significant resourcing was needed to initiate the program of change and investment of this size always requires political will and leadership to identify and enact funding. Building consensus on policy direction and the need for change forms a key part of most system reform where political leadership is required. In divided fields where consensus is not reached and partisan conflict common, politicians tend to stay away [27].

In this case, advocacy by an informal coalition of clinicians, researchers and consumers adopting the same policy position, dovetailed with lobbying support by the lead consumer advocacy body in Australia, The Butterfly Foundation, and the engagement of senior health officials who had supported the development process, all contributed to the policy success. It is often the perception that it is voices of advocacy and lobbying that effect change, but in fact research and analysis has demonstrated that the accretion of consensus and the involvement of senior government officials in particular, is causally linked to the success of policy change movements [28]. The coalescence of expert advocacy and decision-making opinion in the case for reform of the NSW health system to treat EDs, definitively preceded the political leadership decision to move forward with policy change and funding for its implementation. Providing further support for the long-established theory that policy change requires outside “advocates” working with inside “champions” to push change forward [29].

## Principles of the reform

### ***Nothing about me, without me***—***the lived experience voice guides the process***

All reform within this program was based on the principal that there is no one better to diagnose system issues and clarify thinking around remedy than the individuals that have been subject to the pain points. From the outset the project officer employed to scope the sector and models for reform worked alongside a lived experience expert who was also a key member of the advisory committee. Once funded, the agency charged to implement the reform program in NSW, InsideOut Institute (IOI), employed both carer and consumer consultants on staff. All committee’s overseeing the reform at the state and local levels have consumer and carer representatives, as do all guideline and workforce curriculum sub-committees. Care consultation and coordination forms part of the position description of all members of the implementation team—from director through to local area coordinators—who continue to have daily contact with users of the system.

### Generalist versus specialist service model

Both generalist and specialist health service models were considered to underpin the reform. ED treatment delivery in Australia, prior to the reform, had been almost exclusively a job for highly specialised settings—as is the case in many countries around the world. This had resulted in treatment pathways for EDs being dislocated from general health settings, EDs themselves considered almost entirely a specialist concern and excluded from mainstream settings and processes [20]. Health workers within general health settings unsurprisingly reported a lack of knowledge and willingness to treat EDs [22]. Viewed through a service development lens, skills in the treatment of EDs in NSW had become rarefied, exclusively the purview of a very limited number of willing and ‘highly specialised’ health professionals working in a very limited number of settings. People with EDs and their families however, are users of the general health system and present to it for care constantly—to primary care physicians (GPs), to hospitals, clinics, emergency departments and reasonably their expectation is the health system should respond appropriately [14].

The most suitable setting for treatment and management of EDs is also often within general or mainstream health services. People with EDs require long-term medical management, acute medical admissions, longer mental health admissions, brief medical stabilisation, case management—all services offered within the mainstream public health services in NSW and many not offered in the specialist tertiary setting (public or private). Families and carers of people with EDs report often seeing these services as ‘best placed’ to manage their child [14]. Administrative health datasets in NSW confirmed that people with EDs were indeed being treated in ambulatory (outpatient), hospital and emergency department settings all across the state [17], supporting the need for reform within generalist mainstream health services.

When we take into account the relatively high prevalence of EDs in the Australian community [30–32], a health system that syphons off treatment of a relatively common and complex mental and physical condition to a small, specialised, dislocated sector of health, while patients continue to present outside of that sector, across all parts of a system staffed by people who feel unskilled and unwilling to provide service—it is perhaps unsurprising that multiple service and consumer experience issues arise [33].

This is not to say specialist services are not needed. The argument for specialist care requirements for EDs is usually justified by focusing on the complex nature of the illness and the need for high level of skill in both medical and mental health, and usually makes reference to the most severe end of the illness spectrum. Certainly, specialist skills and settings are often required for the containment and treatment of the most severe and complex presentations (NICE guidelines 2017, RANZCP guidelines 2014) and specialist skill and knowledge is needed for the support, upskilling and training of the general health workforce.

For these reasons a hybrid model of service reform was employed in this program, utilising components of both specialist and generalist models of service delivery. Targeted and strategic enhancement of specialist teams, to create genuine specialist hubs at the centre of the system, linked to outreach teams working across the state were employed. These were networked with coordinators, one funded in each health district, to develop general medical and mental health pathways and grow the health workforce for EDs locally.

### Coordination and integration as the targets of reform

How systems of care delivery are structured has major impacts on their relative efficiency and quality of care provided [34]. Poor coordination is considered one of the primary barriers to attaining effective healthcare in many healthcare systems around the world [35]. In recent years, discourse around the organisation and delivery of health and social care services has increasingly focused on the notion of integration [36], particularly in relation to complex and chronic conditions who typically experience fragmented and poorly coordinated management [37, 38]. With the technical definition of chronic illness at one year or greater [39] almost all EDs that enter the health system are classified in this category. Even in those cases where the duration criteria is not met, care is always multidisciplinary, crossing service boundaries, and for the more severe and complex requiring the involvement of multiple services. Across Australian mental health services, coordination and integration remain persistent problems, leading the Productivity Commission in their 2020 report to describe the situation for mental health treatment as an “add-on to the physical health system” with “persistent wasteful overlaps and yawning gaps in service provision” [40]. A systematic review of lived experience of the interface between health sectors identified coordination and communication deficits [41], with other research suggesting care in which coordination of management, a smooth transition between care settings, and shared decision-making are central is valued by those who use the system [42, 43]. Service coordination is also relatively cost effective for governments, often much cheaper than funding for a new clinical program, and in competitive funding environments under constant funding pressure can be appealing for targeted enhancements.

Many have written about the need for integration across mental health care delivery [44] including ‘horizontal’ integration, to bring physical and mental health services together, as well as ‘vertical’ integration in the health system to enable effective partnership between primary, secondary and tertiary mental health services, public, private, federal and state providers [45]. Both were identified as needed in NSW service provision for EDs.

## Methods

### NSW statewide eating disorders service plan

In September 2013, the Mental Health and Drug and Alcohol Office (MHDAO) of the NSW Ministry of Health, launched the *NSW Service Plan for People with Eating Disorders 2013–2018* (‘The Plan’) [46]. The Plan positioned EDs as core business for the NSW Health care system.

It identified the need for prolonged multifaceted integrated care that addresses risks associated with gaps in access to service, underdeveloped pathways for care and sustaining improved outcomes for patients. It outlined the role of Local Health Districts and Specialty Networks (Districts) in improving services for people with EDs. The Service Plan required Districts to make EDs part of the ‘core’ business of their local health service, build capacity and capability to provide evidence-based assessment and treatment, with pathways to options for more intense and specialist treatment to be established at the state level. Phase 1 of the Plan, 2013–2018 received $17.6 million in funding over five years from NSW Health. A second phase was recently funded for a further five years receiving $29.6 million in funding.

The funding for Phase 1 provided for.

- A lead agency to coordinate the implementation of The Plan in NSW (InsideOut Institute)
- A local coordinator in 15 local health district and speciality networks
- An enhancement of the Adult Specialist Tertiary hub at Royal Prince Alfred Hospital in Sydney, to provide nine specialist beds and eight places on a day program and outpatient service
- An enhancement of the Child and Adolescent Specialist Tertiary hub at Children’s Hospital Westmead, to provide 6–10 specialist beds, 8–12 places on a day program alongside their outpatient service
- An Outreach Service at both hubs to provide telemedicine support and training to non-specialist teams in NSW (especially rural and remote teams) providing care to people with EDs
- A statewide workforce training roll-out

### Key strategies of the plan

#### Policy reform at all levels: eating disorders are core business for NSW health

Resistance and the return to homeostasis are expected features of attempts to change any complex system [47]. To prevent the system from returning to status quo a policy directive formed the foundation of the reform program. EDs were to be considered part of the ‘core business’ of the NSW health service. This was outlined in The Plan and was to be reflected in local district policy. NSW is over three times the geographical size of the UK and divided into local health districts (some called speciality networks). Over the first two years of The Plan the coordinating agency for implementation—InsideOut Institute—undertook an engagement period with districts to educate them on the nature of The Plan, the policy directive, and establish buy-in to the reform program. The first key target of the implementation was achieved by the end of the second year—each of the 15 districts/networks had accepted EDs are core business for their district and had ratified a local policy and plan for change.

#### Whole of system change: eating disorders are a whole of health issue

While the development of The Plan was driven by the Mental Health Branch within NSW Ministry of Health, the decision was taken prior to the release of The Plan to undertake the extra processes to release it as a whole-of-health plan. This required review and endorsement at senior levels across the health department but meant that The Plan had the legitimacy and buy-in from all sectors of health administration.

This decision was both strategic and practical, as the targets of change spanned the whole health system including emergency departments, general medical and mental health hospital wards, mental health community treatment teams. As others have observed [1] whole of system change to drive integrated service reform requires commitment from the most senior levels of management, the recalibration of resources across the system, the involvement of many levels of existing management and staffing, and the redefining of key worker functions and roles. These are not processes that can be ‘scaled up’ to from any single project, they need derive from a whole of system strategy.

#### Governance at every level

**Fig. 1:**
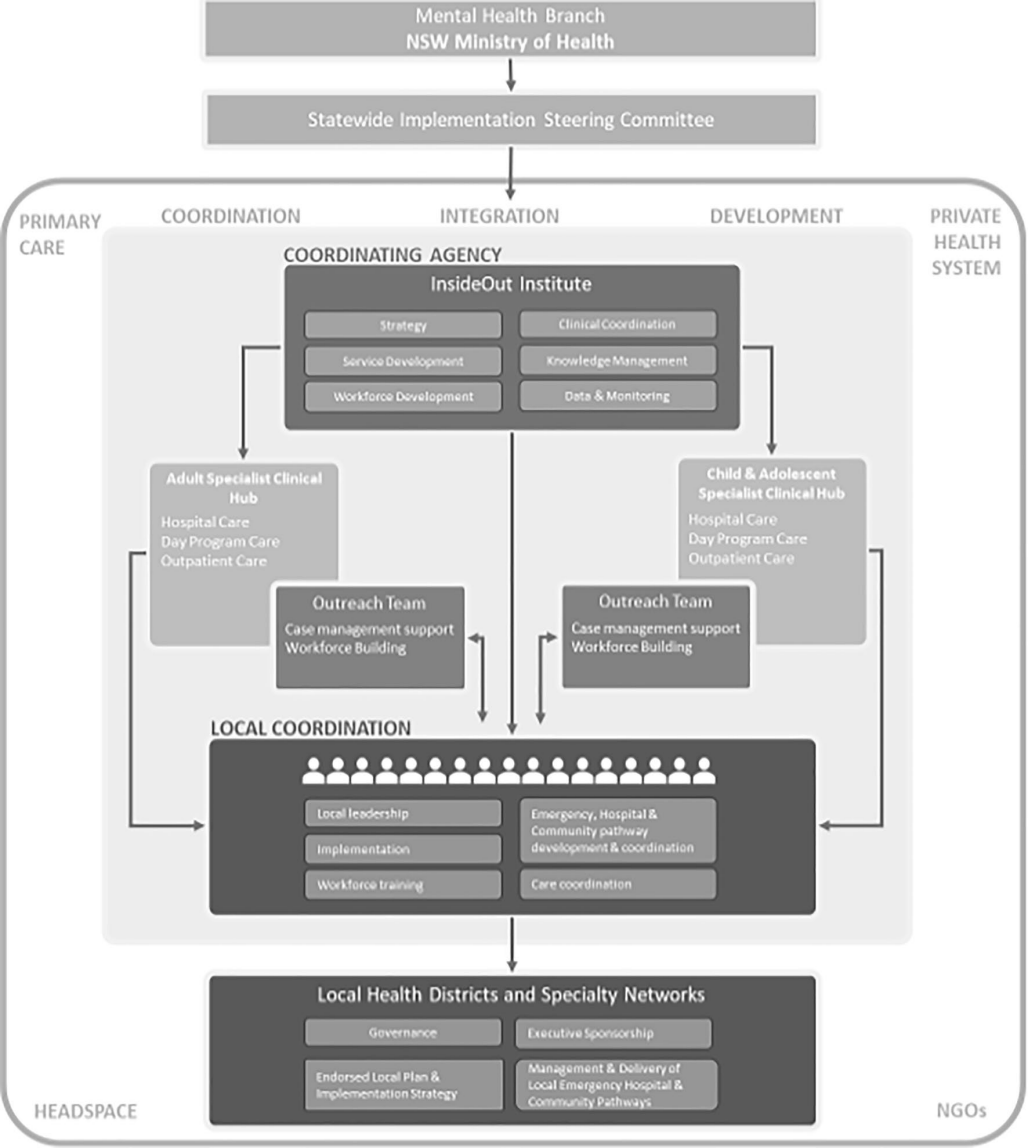
Governance structure for the Statewide plan

As is depicted in Fig. 1, the reform program has a layered governance structure that reflects the governance and levels of responsibility of the health system in the jurisdiction. In NSW districts and networks receive funding from NSW Health but govern as autonomous units in terms of care delivery. Chief Executives of Districts are managed according to Key Performance Indicators (KPIs) set by NSW Health while managing their own budgets and services and also reporting to local boards. Hence governance structures needed to be multi-level and linked.

Strong local governance, connected to statewide governance structures with both local and statewide support structures connected to and working with the governance teams were employed. All participating Districts have established a steering committee and developed a Local Plan that reflects the aims and strategies of The Plan. Executive leadership is strongly evident in local governance structures for EDs, with Chief Executives the sponsor of The Plan in most locations and a major role for Mental Health Directors and Medical Leads.

#### Collaboration and staged implementation

As a whole of system reform, it was accepted that the process of change would be gradual, involving close to continuous collaboration between stakeholders at all levels including government, senior health bureaucrats, lived experience users of the system, frontline staff, service providers, and the implementation team [48]. Hence, while the process would be staged, an implementation plan produced and signed-off at all levels, and governance for the project led by the health department, the implementation itself was driven by collaboration, resembling more at times an integrated and evolving process than distinct inflexible stages.

#### Coordination and integration

One of the primary targets of the program of reform is the coordination and integration of service elements, staff and skills to develop effective, consistent and continuous pathways to care that alter the patient experience and outcomes. Local coordinators were funded in Phase 1 within districts in acknowledgment of the importance of this key strategy. Their role is very broad, including clinical and operational change components. They work across rather than within team and district structures. Broadly their work tasks fall into three categories:

- service development (working across all levels of the district and with service providers outside of the state system) to build pathways to care;
- workforce development (working across all sectors of health within the district and within service partners outside of state health) to grow the workforce capacity and capability; and
- case coordination and care planning for people with EDs receiving care within the district

A coordinating agency, IOI, was also engaged to lead and coordinate the implementation of The Plan. A key strategy of the statewide Implementation Plan is to provide integrated support across the state. IOI provide central guidance, templates, implementation support, and monitoring of the development of local service plans, develop and evaluate centrally every workforce development program that the coordinators roll-out locally, integrate the roles of the tertiary hubs, outreach teams and coordinators to form a statewide network of expertise and support, and provide support structures, resources, consultation and documents to support Local Health Districts (LHDs) and Speciality Networks (SNs) to implement their Local Service Plans and enhance their local capacity.

IOI report to NSW Health on their implementation of The Plan and also act as an expert resource to the health department on ED matters, providing information and advice for planning and management purposes.

#### Building specialist hubs at the centre of the network

A hub and spoke model of service development informed the program. A targeted and strategic use of resources ensured an increase in tertiary services for all age groups, to provide inpatient, day-program and outpatient continuum care hubs for both Adults (at RPA Hospital) and Children and Adolescents (at Children’s Hospital Westmead). These function to admit the most unwell and complex patients for care, and as a point of escalation for district and their coordinators when all endeavours to manage the treatment needs of the patient locally have been exhausted. Outreach services were funded at both the Adult and Child & Adolescent Tertiary Specialist Hubs to provide direct telemedicine support to local teams providing care to people with EDs, to enhance the ability to manage people locally wherever possible, and to developing the capability and confidence of health professionals.

#### Local treatment pathway development

The Plan outlined clearly the service targets, and district service plans needed to address each of these in their strategic and implementation documents. There are three major service targets, each involves the development of a series of procedures and protocols, the re-arrangement and at times procurement of services as well as significant development of the workforce. They are:

- open and develop emergency department pathways;
- ensure critically unwell patients are being admitted to hospital (both medical and mental health sectors); and
- increase treatment in the community (community mental health teams a primary target)

#### Workforce development

Growing and developing the workforce underpins all strategies, activities and goals of The Plan. The nature of the reform across a large geographic region involving metropolitan centres, many regional centres and vast expanses of rural and remote communities required a blended approach to workforce training. A particular challenge was engaging and reaching the health workforce dispersed across the geographies to deliver training to them from the central coordinating agency (IOI). The workforce training program as a result relied heavily on the development of evidence-based online training programs, accompanied by face-to-face intensive workshops delivered at the most major regional or metro centre within each of the districts. Ongoing supervision for up to 12 months for more complex skills (e.g. delivery of manualised evidence-based therapies) was delivered by telehealth into most locations as part of the workforce model to ensure skills translated into practice.

IOI worked closely with the local coordinators and Outreach Teams to develop and implement a program of workforce training to ensure that the health workforce even in the most remote and isolated areas were reached and engaged within the program.

Workforce development was built into all Local plans as a strategic priority.

The following five priority areas were identified and delivered in Phase 1 of the implementation:

- screening, identification and diagnosis
- medical management in the community
- hospital treatment
- clinical management and case management
- delivering evidence-based treatments within the community

#### Data and monitoring

A systematic review of governance models in reform identified a number of strategies common to service development processes aiming for integration of services in health systems, and one of those common to almost all is data and monitoring for quality improvement [36]. This reform program utilises data at all levels of governance and reporting. Dashboards for the monitoring of key deliverables for implementation targets are used at both the statewide and local steering committee levels. De-identified linked emergency department, hospital and community data for every person in NSW diagnosed (either primary or secondary) with an ED receiving care in the system has been accessed for every year of The Plan and the two years prior, to monitor change in occasion of service and continuum of care as pathways develop. District level data on these occasions of service in all three service settings (emergency, hospital and community) is distributed back to districts quarterly to drive change locally. Analyses of the findings for publication on the first five years of the implementation data and process is currently being prepared.

## Conclusions

An external review of Phase 1 of The Plan’s implementation has demonstrated an effect of the policy change on all three service targets—emergency departments, hospitals and community [25]. Occasions of service for people with EDs in all three service settings increased over the first five years of implementation (2013–2018) indicating that pathways are opening and EDs are being seen as part of the core business of the health system. The report documented particular change in the culture and attitudes of senior health officials and frontline staff to the treatment of people with EDs and the role of the public health system, concluding that much progress had been made on the acceptance of EDs as core business. Multiple strengths of the governance model were identified, with district executive leadership on local steering committees seen as a particular strength of governance arrangements for local service plans. The evaluation found widespread executive support for the recognition of EDs as core business which was a dramatic change from pre-plan. As well as pathway development and increased rates of treatment provision across districts, more micro-level changes reported in the evaluation included improved communication between emergency department, medical teams, allied health and psychology, often facilitated by the EDs coordinator; greater interaction between paediatric and adult medical teams; regular meetings between paediatric and Child and Adolescent Mental Health Service (CAMHS) teams; and regular case conferences for admitted patients.

The report identified three key enablers of the success of the change process to date (see summary table below). Executive sponsorship and leadership—the influence of engaged leadership was seen as a key factor in the progression of Local Plans. The local coordinator positions—having someone skilled in EDs in a central role, able to provide support across the LHD was viewed as a strong enabler. The central coordinating agency (InsideOut)—the provision of centralised advice and support, networking and visiting local sites, representation and consistency across all working parties, and the provision of standardised training across the state to build the health workforce were all seen as key parts of the success of this element of the governance and implementation structure.

All service change is by nature location specific however there are shared elements in any complex health system, and commonalities to the process of change management in human led organisations. While this large-scale reform is specific in some ways to the Australian health system context it utilised well documented processes to initiate the policy reform (Table 1), and strategies well known to health systems that may well be useful in other contexts (Table 2), and also identified a number of key enablers to successful change (Table 3) that could be considered in other settings and countries to achieve similar outcomes. These are summarised in table form for ease of translation.

**Table 1:** Key factors for policy reform

**Key factors for policy reform**
*Lived experience informed*: those with lived experience lead reform planning and targets and voice the imperative
*Building consensus & advocating*: stakeholders voices supporting the same broad policy position, and the engagement of senior bureaucrats
*Political will and leadership*: a political ‘sponsor’ or supporter with access to the right levers needs to be engaged
*Adequate resourcing*: funding for a full program of change needs be achieved usually through the political advocate
*Time*: real policy and practice change takes time, is iterative and ongoing

**Table 2:** Key Strategies utilised in this reform

**Key strategies in this reform**
A policy directive to prevent the system from returning to status quo
Whole of System Change rather than single program change, with buy-in across all levels of the system
Governance for implementation at all levels with accountabilities, so it is genuinely whole of system
Continuous collaboration between stakeholders to bring about change in stages
Coordination and Integration key targets
Funding of Specialist hubs with ‘spokes’ out to the districts
Local access and treatment pathway development across all settings
Workforce development rolled out at large scale
Data and Monitoring of implementation and service parameters continuously

**Table 3:** Identified key enablers

**Key enablers identified**
Executive leadership at the local level
A skilled local coordinator to ‘own’ and guide the process
A coordinating agency for centralised support and resourcing

This reform is very large in scale and is now in its second Phase of implementation. The next iteration of The Plan has just been launched [49]. In phase 2 all three enablers identified in the review have been maintained and enhanced. All local coordinator positions have been increased to full-time, the coordinating agency received an increase in funding and positions, and the engagement of the executives in local and statewide processes continues.

The data supports the 15-year time frame it is anticipated the program will need to create broad, sustainable change to all service pathways and more importantly the patient and carer experience within those pathways.

Like all successful large-scale reforms of complex systems this one is viewed as an iterative, evolving and ultimately continuous process that will for some years require intensive investment of time and resources before sustainable change has been reached and the new normal established. There is plenty more work to be done to ensure that every person with an ED and their families receive timely access to evidence-based care in this part of Australia.

## Data Availability

Not applicable.

## Abbreviations

NSW: New South Wales
CAMHS: Child and Adolescent Mental Health Services
ED: Eating disorder
GP: General practitioner
IOI: InsideOut Institute
KPI: Key performance indicator
LHD: Local Health District
MHDAO: Mental Health Drug and Alcohol Office
MP: Member of Parliament
NICE: National Institute for Health and Care Excellence
RANZCP: Royal Australian and New Zealand College of Psychiatrists
SN: Statewide network
